# Investigations of Potential Phenotypes of Foot Osteoarthritis: Cross‐Sectional Analysis From the Clinical Assessment Study of the Foot

**DOI:** 10.1002/acr.22677

**Published:** 2016-01-25

**Authors:** Trishna Rathod, Michelle Marshall, Martin J. Thomas, Hylton B. Menz, Helen L. Myers, Elaine Thomas, Thomas Downes, George Peat, Edward Roddy

**Affiliations:** ^1^ Arthritis Research UK Primary Care Centre, Research Institute for Primary Care & Health Sciences, Keele UniversityKeeleStaffordshireUK; ^2^ Arthritis Research UK Primary Care Centre, Research Institute for Primary Care & Health Sciences, Keele University, Keele, Staffordshire, UK, and Lower Extremity and Gait Studies Program, School of Allied Health, La Trobe UniversityBundooraVictoriaAustralia

## Abstract

**Objective:**

To investigate the existence of distinct foot osteoarthritis (OA) phenotypes based on pattern of joint involvement and comparative symptom and risk profiles.

**Methods:**

Participants ages ≥50 years reporting foot pain in the previous year were drawn from a population‐based cohort. Radiographs were scored for OA in the first metatarsophalangeal (MTP) joint, first and second cuneometatarsal, navicular first cuneiform, and talonavicular joints according to a published atlas. Chi‐square tests established clustering, and odds ratios (ORs) examined symmetry and pairwise associations of radiographic OA in the feet. Distinct underlying classes of foot OA were investigated by latent class analysis (LCA) and their association with symptoms and risk factors was assessed.

**Results:**

In 533 participants (mean age 64.9 years, 55.9% female) radiographic OA clustered across both feet (*P* < 0.001) and was highly symmetrical (adjusted OR 3.0, 95% confidence interval 2.1, 4.2). LCA identified 3 distinct classes of foot OA: no or minimal foot OA (64%), isolated first MTP joint OA (22%), and polyarticular foot OA (15%). After adjustment for age and sex, polyarticular foot OA was associated with nodal OA, increased body mass index, and more pain and functional limitation compared to the other classes.

**Conclusion:**

Patterning of radiographic foot OA has provided insight into the existence of 2 forms of foot OA: isolated first MTP joint OA and polyarticular foot OA. The symptom and risk factor profiles in individuals with polyarticular foot OA indicate a possible distinctive phenotype of foot OA, but further research is needed to explore the characteristics of isolated first MTP joint and polyarticular foot OA.

## INTRODUCTION

The pattern and location of joint involvement have played a fundamental role in shaping the current understanding of osteoarthritis (OA). Whether it is the differing effects of risk alleles and gene expression on hip and knee OA 1, the contrasting risk profiles of tibiofemoral and patellofemoral joint OA 2, or the symmetry and clustering of small joint involvement in hand OA 3, joint‐specific perspectives have proved insightful.

Box 1 **Significance & Innovations**

- There is a lack of epidemiologic studies investigating the patterning of foot osteoarthritis (OA).
- We present first empirical evidence for the separation of first metatarsophalangeal joint OA from a form of multijoint “polyarticular foot OA” on the basis of patterning of joint involvement on plain radiographs.
- The symptom and risk factor profiles of those with polyarticular foot OA indicate a possible distinct phenotype of foot OA.

The foot joint complex presents a novel challenge in this regard. With few exceptions, population‐based epidemiologic studies have focused on the metatarsophalangeal (MTP) joints, predominantly the first MTP, for the purpose of estimating prevalence 4. Using a recently developed radiographic atlas for semiquantitative scoring of plain radiographs of the feet 5, Menz et al 6 and Roddy et al 7 have observed the frequent occurrence of osteophytes or joint space narrowing (JSN) in joints located in the medial column of the midfoot (specifically, the second cuneometatarsal [CM] joint, talonavicular [TN] joint, and navicular first cuneiform [NC] joints). While it remains the case that the first MTP joint is most commonly implicated in foot OA, these observations could be consistent with 2 quite different scenarios, both of which carry implications for how foot OA is understood and managed: either there are forms of OA at the foot that occur independently, or first MTP joint OA is associated with OA at other proximal joints in the foot as part of a more widespread polyarticular presentation.

Distinctions in the patterning and risk factor profiles of foot OA have the potential to provide new insights into causation. The foot may be similar to the hand in that specific localized risk factors could be associated with limited forms of OA, while systemic risk factors, including age, sex, and metabolic factors are more likely to be associated with more widespread polyarticular forms of OA. The accompanying symptoms may also vary in different forms of foot OA. The identification of phenotypes at other sites, such as the thumb base and patellofemoral joint, has led to greater understanding about the etiology and presentation of OA at these locations 8, 9. Early research targeting treatments for these sites has shown some positive outcomes 10, 11, and this approach may also be appropriate for different forms of foot OA.

In this study, we sought to analyze cross‐sectional data from a population‐based survey of foot pain and OA in adults ages ≥50 years to investigate patterns of radiographic foot OA through examination of clustering, symmetry, and co‐occurrence of joint involvement in the foot. Latent class analysis (LCA) was used to determine whether subgroups of foot OA existed, and these were compared with respect to their symptom and risk factor profiles.

## PATIENTS AND METHODS

#### Study design

The Clinical Assessment Study of the Foot (CASF) is a prospective observational cohort study. All adults ages ≥50 years registered with 4 general practices in North Staffordshire, UK, were mailed a health survey questionnaire, irrespective of any foot‐related health care consultation. Responders to the health survey reporting pain in or around the foot within the last year and who consented to further contact were invited to attend a research clinic 12. A flowchart showing the recruitment of participants to the CASF study has been published previously 7.

All participants provided written informed consent. Ethical approval for the study was obtained from the Coventry Research Ethics Committee (reference number 10/H1210/5).

#### Scoring of foot radiographs

At the research clinics, weight‐bearing dorsoplantar and lateral radiographs were taken separately of each foot, according to a standardized protocol. A single experienced reader (MM), who had undergone a period of training, scored 5 joints in each foot (first MTP joint, first and second CM joints, NC joint, and TN joint) for osteophytes and JSN (range 0–3) according to a published atlas 5. The joints examined were selected based on their inclusion in the published radiographic foot atlas, which had determined that they were the most commonly affected, clearly visible on dorsoplantar and lateral views, and could be reliably scored 5. Sixty randomly selected radiographs were rescored after 8 weeks (by MM) to assess intrarater reliability and were scored by a second experienced reader (HBM) to determine interrater reliability. As reported previously, reliability for the presence of OA was excellent for intrarater (mean κ = 0.94; mean % exact agreement 99%) and moderate for interrater reliability (mean κ = 0.46; mean % exact agreement 79%) 7.

Radiographic OA in a foot joint was defined as grade ≥2 for osteophytes or JSN on either dorsoplantar or lateral views.

Individuals were excluded from the current analyses if medical records (primary care or local hospital) or a clinical radiology report by a consultant musculoskeletal radiologist identified them as having rheumatoid, psoriatic, or nonspecific inflammatory arthritis.

#### Descriptive characteristics and symptoms

The following information was collected in the health survey questionnaire: higher education attendance, foot pain location by foot, foot pain in the first MTP joint and midfoot regions as indicated on a foot manikin 13, foot pain duration, number of days with foot pain, aching or stiffness in the last month 14, foot pain severity by numerical rating scale (range 0–10), satisfaction with foot symptoms, Manchester Foot Pain and Disability Index (MFPDI) 15, Short Form 12 physical and mental component scores 16, and Hospital Anxiety and Depression Scale 17. Further details on the data collection methods and outcome measures can be found in the published study protocol 12.

#### Risk factor profiles

A number of potential risk factors previously found to be associated with foot OA were examined, including age, sex, obesity, and structural characteristics (hallux valgus, footwear, and previous foot/ankle injury) 18. In addition, metabolic factors (hypertension, type 2 diabetes mellitus, impaired fasting glucose [IFG], dyslipidemia, and lipid‐lowering drugs) and nodal OA, which have been implicated in OA etiology at other joints, were investigated 19, 20, 21, 22. Demographic data (age, sex, occupation), along with the presence of hip pain and knee pain in the last year, wearing of high‐heeled and narrow‐toed footwear between the ages of 20 and 49 years, 12, and intermittent claudication determined from the Edinburgh Claudication Questionnaire 23, were collected in the health survey questionnaire. Self‐reported hallux valgus was determined using a validated line‐drawing instrument consisting of 5 drawings for each foot, with each one illustrating a sequential increase in hallux valgus angle of 15° 24. Participants selected the drawing that best depicted the severity of hallux valgus for each foot. Hallux valgus was classified as present in a foot if any of the 3 most severe drawings were selected 24. At the research clinics, the presence of finger nodes were determined by observation and palpation, and height and weight were measured to calculate body mass index (BMI). Previous foot and ankle injuries were recorded during a standardized clinical interview 12. Posteroanterior radiographs were also taken of each hand and interphalangeal joints were scored (by MM) for the presence of OA (Kellgren/Lawrence grade ≥2). Primary care medical records were reviewed for participants providing consent (95%). Diagnoses or consultations for hypertension, type 2 diabetes mellitus or IFG, and dyslipidemia (raised cholesterol or triglycerides) or a prescription of a lipid‐regulating drug in the 18 months prior to clinic attendance were identified. A classification of metabolic syndrome was defined as the presence of ≥3 of the following: BMI ≥30 kg/m^2^, hypertension, dyslipidemia, and type 2 diabetes mellitus or IFG (based on previous criteria) 25.

#### Statistical analysis

Clustering of joint involvement within the foot was examined using the chi‐square test, with the expected frequency calculated from the Poisson distribution. The frequency of OA in a joint occurring in isolation and with other joints in the same foot was calculated. Logistic regression was used to examine the interrelationships of radiographic OA at different pairs of joints within each foot and the presence of symmetrical radiographic OA affecting the same joint in both feet. Generalized estimating equations were used to determine overall symmetry across the 5 foot joints, adjusting for age, sex, presence of OA in each foot joint, and the number of foot joints affected with radiographic OA within the person. Results are presented as odds ratios (ORs) with 95% confidence intervals (95% CIs).

LCA was undertaken to identify classes of radiographic foot OA based on the presence of radiographic OA in the joints of the feet. The optimal number of classes was determined by a combination of the following: 1) goodness‐of‐fit statistics (Akaike Information Criteria, Bayesian Information Criteria [BIC], sample size–adjusted BIC, and the Lo‐Mendell‐Rubin adjusted likelihood ratio test [LRT]) 26; 2) uncertainty of classification measures (entropy 27 and average posterior probabilities 28); 3) class size of at least 10% of the sample; and 4) clinical relevance and interpretability.

Further investigation to compare the descriptive characteristics, symptoms, and risk factor profiles of each of the classes of foot OA identified by LCA was undertaken. Analyses were adjusted for age and sex, which were considered potential confounders. For continuous data, multiple linear regression was used; means and their 95% CIs were presented for each latent class, with significant differences between the classes being determined using F tests. For dichotomous and ordinal data, logistic regression was used to obtain probabilities and their 95% CIs; significant differences between the classes were established using chi‐square tests. With regard to the MFPDI, scores have previously been shown to fit the Rasch model, and this form was used for both subscales 29.

All analyses were 2‐tailed and were deemed statistically significant if the *P* value was less than 0.05. Analysis was performed using Stata, version 13, except the LCA, which was performed in Mplus, version 7.11 30.

## RESULTS

Of the 560 participants who attended research clinics, 24 with inflammatory arthritis were excluded and 3 did not have foot radiographs, leaving 533 for analysis. Participants had a mean ± SD age of 64.9 ± 8.4 years and 55.9% were female. Radiographic data were missing for 12 first MTP joints, affecting 8 participants. Overall, 62.7% had radiographic OA in ≥1 foot joints, with the first MTP joint being the most frequently affected (27%, n = 287) followed by the second CM joint (17%, n = 184), TN joint (15%, n = 158), NC joint (8%, n = 86), and the first CM joint (5%, n = 50) (see Supplementary Figure 1, available on the *Arthritis Care & Research* web site at http://onlinelibrary.wiley.com/doi/10.1002/acr.22677/abstract).

The mean ± SD number of joints affected was 1.4 ± 1.6, with 21% of participants having OA in 1 joint and 42% having OA in ≥2 joints (Table 1). Radiographic OA was found to cluster significantly in individuals across both feet (*P* < 0.001), more than was expected by chance, but clustering was not seen separately in the left (*P* = 0.078) or right foot (*P* = 0.575) (Table 1). The analysis was repeated stratifying by sex and the same findings occurred in both males and females, although females had slightly higher frequencies of joint involvement (data not shown).

**Table 1:** Observed and expected numbers of joints with radiographic OA in the feet of 533 adults ages ≥50 years^a^

Joints with radiographic OA, no.	Left foot, 0–5 (n = 533)			Right foot, 0–5 (n = 533)			Across both feet, 0–10 (n = 533)		
	Observed	Expected	Significance	Observed	Expected	Significance	Observed	Expected	Significance
0	280 (52.5)	269		259 (48.6)	252		199 (37.3)	127	
1	164 (30.8)	184		182 (34.2)	189		113 (21.2)	182	
2	67 (12.6)	63		65 (12.2)	71		111 (20.8)	131	
3	21 (3.9)	14		21 (3.9)	18		54 (10.1)	63	
4	1 (0.2)	2		5 (0.9)	3		29 (5.4)	22	
5	0 (0)	0		1 (0.2)	0		14 (2.6)	6	
6	b	b		b	b		9 (1.7)	2	
7	b	b		b	b		4 (0.8)	0	
8	b	b		b	b		0 (0)	0	
9	b	b		b	b		0 (0)	0	
10	b	b		b	b		0 (0)	0	
Chi‐square value			6.8			2.9			161.2
df			3			4			6
*P*			0.078			0.575			< 0.001

a Values are the number (percentage) or number unless indicated otherwise.

b Only 5 joints in each foot were assessed.

Radiographic OA in the first MTP joint tended to occur in isolation, whereas OA in the NC joint, second CM joint, and first CM joint tended to co‐occur with other joints (Table 2). When stratified by sex the same findings were seen except for first CM joint OA, which occurred slightly more frequently in isolation in males compared to females (data not shown). The possible combinations of joint involvement are presented in a 5‐way Venn diagram in Supplementary Figure 2 (available on the *Arthritis Care & Research* web site at http://onlinelibrary.wiley.com/doi/10.1002/acr.22677/abstract).

**Table 2:** Frequency of radiographic OA occurring in isolation and combined with other joints in the same foot, in 1,066 feet^a^

Foot joint	Frequency of radiographic OA occurring in isolation from other joints in the same foot	Frequency of radiographic OA occurring with ≥1 other joint in the same foot
First MTP joint	60.6 (174/287)	39.4 (113/287)
First CM joint	40.0 (20/50)	60.0 (30/50)
Second CM joint	33.7 (62/184)	66.3 (122/184)
NC joint	17.4 (15/86)	82.6 (71/86)
TN joint	47.5 (75/158)	52.5 (83/158)

a Values are the percentage (number/total number). OA = osteoarthritis; MTP = metatarsophalangeal; CM = cuneometatarsal; NC = navicular first cuneiform; TN = talonavicular.

Although unilateral OA was more prevalent than bilateral OA, strong associations were seen for symmetry in each of the foot joints, with the strongest association found in the NC joint, where the odds of NC joint OA, given its presence in the same joint in the other foot, increased 20‐fold (Table 3). The unadjusted overall symmetry for foot OA was OR 12.9 (95% CI 9.9, 16.8). After adjustment for age, sex, presence of OA in each foot joint, and the total number of foot joints with radiographic OA across both feet, the OR for overall symmetry remained significant but was reduced to 3.0 (95% CI 2.1, 4.2). This indicates the presence of confounding; sensitivity analysis found that the total number of foot joints with radiographic OA across both feet was the variable that caused the largest reduction in the odds. Stratification by sex produced similar results, but overall adjusted symmetry in the foot was stronger in females (OR 4.3 [95% CI 2.7, 6.8]) compared to males (OR 1.8 [95% CI 1.1, 3.1]).

**Table 3:** Symmetry of radiographic OA in the left and right feet of 533 adults ages ≥50 years^a^

Foot joint	Individuals examined, total no.	No OA in either foot, no. (%)	OA in left foot only, no. (%)	OA in right foot only, no. (%)	OA in both left and right feet, no. (%)	OR (95% CI)^b^
First MTP joint	525	329 (62.7)	45 (8.6)	62 (11.8)	89 (17.0)	10.5 (6.7, 16.5)
First CM joint	533	490 (91.9)	23 (4.3)	13 (2.4)	7 (1.3)	11.5 (4.2, 31.5)
Second CM joint	533	397 (74.5)	38 (7.1)	50 (9.4)	48 (9.0)	10.0 (6.0, 16.8)
NC joint	533	468 (87.8)	19 (3.6)	25 (4.7)	21 (3.9)	20.7 (9.9, 43.3)
TN joint	533	414 (77.7)	35 (6.6)	45 (8.4)	39 (7.3)	10.3 (5.9, 17.8)

a OA = osteoarthritis; OR = odds ratio; 95% CI = 95% confidence interval; MTP = metatarsophalangeal; CM = cuneometatarsal; NC = navicular first cuneiform; TN = talonavicular.

b The odds of having OA in a joint given its presence in the same joint in the other foot.

**Figure 1:**
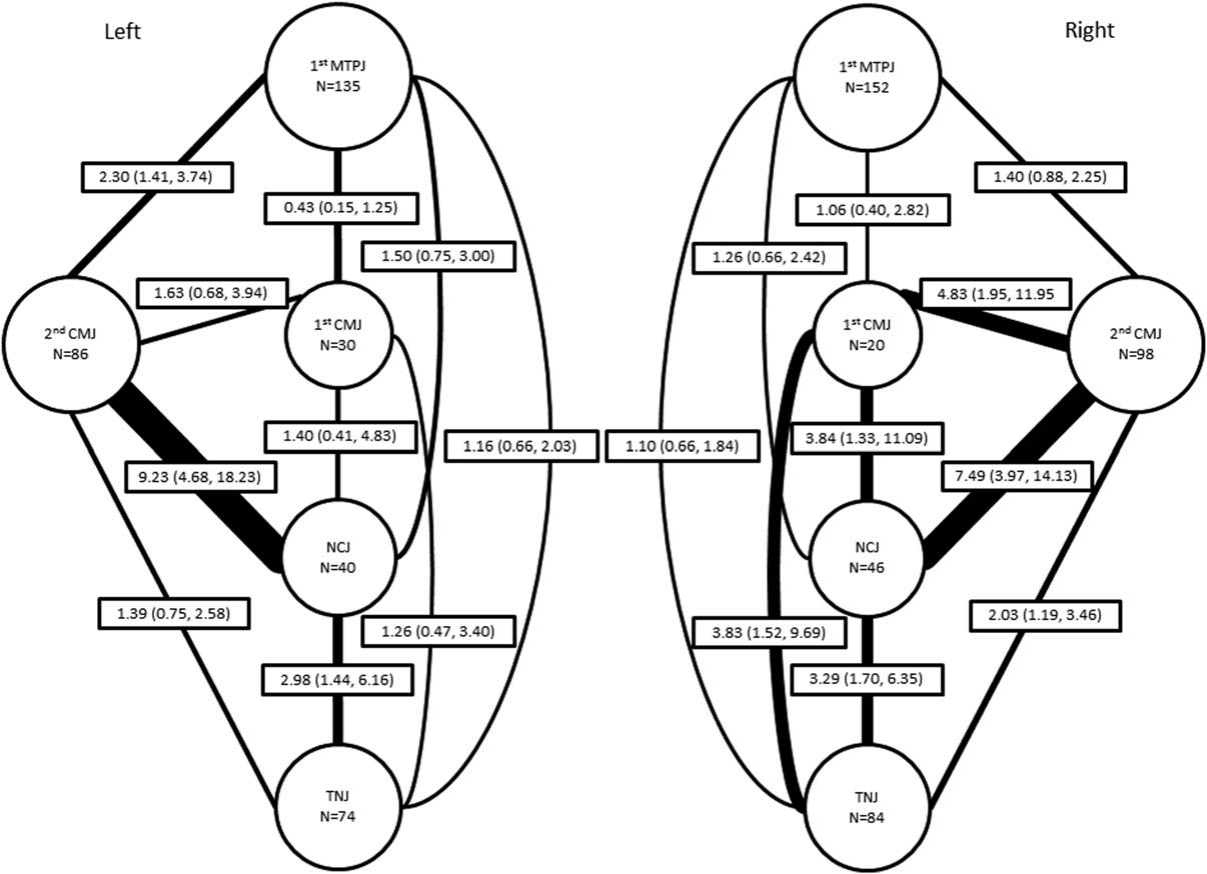
Node and edge diagram showing the frequency of osteoarthritis (OA) and the association of radiographic OA between pairs of joints of the left foot and right foot of 533 adults ages ≥50 years. The size of each node is proportional to the frequency of OA in that joint and the width of the edge is proportional to the odds ratio between each pair of joints. MTPJ = metatarsophalangeal joint; CMJ = cuneometatarsal joint; NCJ = navicular first cuneiform joint; TNJ = talonavicular joint.

Bivariate associations between paired combinations of foot joints within the left foot were found to be statistically significant between the second CM joint and NC joint, the NC joint and TN joint, and the first MTP joint and second CM joint (Figure 1). In the right foot, statistically significant associations were found between all paired combinations of the midfoot joints (first CM joint, second CM joint, NC joint, and TN joint) (Figure 1).

LCA of radiographic OA in each foot joint was undertaken (Supplementary Table 1, available on the *Arthritis Care & Research* web site at http://onlinelibrary.wiley.com/doi/10.1002/acr.22677/abstract). The 3‐class solution was considered the best fit as the BIC was at its lowest, the Lo‐Mendell‐Rubin–adjusted LRT indicated that the 4‐class solution was not significantly better than the 3‐class solution, and entropy was high. The 3‐class solution also had average posterior probabilities that were >0.7 (Table 4), indicating better classification and greater distinction between latent classes compared to the other class solutions, and all classes were at least 10% of the total sample.

**Table 4:** Latent classes of radiographic foot OA^a^

	**Class 1: no or minimal foot OA**	**Class 2: isolated first MTP joint OA**	**Class 3: polyarticular foot OA**
Class size (%) based on most likely latent class membership	341 (64.0)	115 (21.6)	77 (14.5)
Average posterior probabilities for most likely latent class membership	0.969	0.937	0.844
Right first MTP joint	0.130	0.723	0.348
Right first CM joint	0.016	0.013	0.162
Right second CM joint	0.061	0.205	0.665
Right NC joint	0.031	0.000	0.436
Right TN joint	0.118	0.152	0.329
Left first MTP joint	0.000	1.000	0.310
Left first CM joint	0.049	0.031	0.119
Left second CM joint	0.052	0.152	0.627
Left NC joint	0.013	0.032	0.392
Left TN joint	0.110	0.134	0.265

a Values are probabilities unless indicated otherwise. OA = osteoarthritis; MTP = metatarsophalangeal; CM = cuneometatarsal; NC = navicular first cuneiform; TN = talonavicular.

In the 3‐class solution, class 1 was the largest (n = 341, 64%) and was characterized by low probabilities of radiographic OA occurring in all 10 foot joints, and was therefore labeled as “no or minimal foot OA.” Class 2 (n = 115, 22%) had high probabilities of radiographic OA in the first MTP joint in both the left and right feet, and was labeled as “isolated first MTP joint OA.” Class 3 (n = 77, 15%) had medium‐to‐high probabilities of OA in both second CM joints and NC joints in the midfoot with medium probabilities of OA in the TN joints and first MTP joints, and therefore was labeled as “polyarticular foot OA” (Table 4).

The isolated first MTP joint OA and polyarticular foot subgroups were significantly older than the no or minimal foot OA subgroup, after adjustment for sex (Table 5). Following adjustment for age, the polyarticular foot subgroup had a significantly higher probability of being female in comparison to the other subgroups. After adjustment for both age and sex, the polyarticular foot OA subgroup had significantly more persistent and severe pain, greater functional limitation, higher BMI, and increased presence of nodal hand OA compared with the other subgroups. No statistically significant between‐group differences were seen for socioeconomics, recalled footwear at ages 20–49 years, previous foot/ankle injury, and selected metabolic factors.

**Table 5:** Characteristics and risk factors of the 3 distinct classes of foot OA identified by latent class analysis adjusted for age and sex^a^

	No or minimal foot OA^b^	Isolated first MTP joint OA^b^	Polyarticular foot OA^b^	Difference between the 3 groups (significance)^b^
Frequency, no. (%)	341 (64.0)	115 (21.6)	77 (14.5)	–
Descriptive characteristics and symptoms				
Duration of foot pain				0.172
<12 months	48; 0.14 (0.11, 0.17)	15; 0.11 (0.07, 0.15)	6; 0.11 (0.06, 0.15)	
1 to <5 years	120; 0.33 (0.28, 0.37)	28; 0.29 (0.23, 0.34)	18; 0.28 (0.22, 0.35)	
5 to <10 years	63; 0.23 (0.19, 0.26)	29; 0.23 (0.20, 0.27)	30; 0.23 (0.20, 0.27)	
≥10 years	110; 0.30 (0.26, 0.35)	43; 0.37 (0.29, 0.45)	23; 0.38 (0.28, 0.47)	
Foot pain on most or all days in last month	171; 0.50 (0.45, 0.56)	57; 0.51 (0.41, 0.60)	51; 0.69 (0.59, 0.79)	0.012
Foot pain in both feet	166; 0.50 (0.44, 0.55)	55; 0.47 (0.38, 0.56)	51; 0.65 (0.54, 0.76)	0.046
First MTP joint foot pain in last month	140; 0.41 (0.36, 0.47)	57; 0.51 (0.42, 0.60)	52; 0.69 (0.59, 0.80)	< 0.001
Midfoot pain in last month	175; 0.52 (0.47, 0.58)	47; 0.41 (0.32, 0.50)	52; 0.69 (0.59, 0.80)	0.001
Very or somewhat dissatisfied with foot symptoms	168; 0.49 (0.44, 0.54)	47; 0.43 (0.34, 0.53)	45; 0.61 (0.50, 0.73)	0.054
Foot joint radiographic OA grade ≥2 (0–10), mean no. (95% CI)	0.6 (0.5, 0.7)	2.4 (2.2, 2.6)	3.8 (3.4, 4.1)	< 0.001
Foot pain severity (0–10) in last month, mean (95% CI)	5.2 (5.0, 5.5)	4.9 (4.4, 5.4)	6.0 (5.4, 6.6)	0.020
MFPDI (5‐point scale: −2 to 2), mean (95% CI)^c^				
Pain subscale	−0.3 (−0.4, −0.1)	−0.5 (−0.7, −0.2)	0.3 (0.0, 0.7)	0.002
Function subscale	−0.7 (−1.0, − 0.5)	−0.9 (−1.3, −0.5)	0.0 (−0.4, 0.5)	0.007
SF‐12, mean (95% CI)^d^				
Physical component score	38.0 (36.6, 39.3)	40.3 (38.1, 42.5)	37.2 (34.2, 40.1)	0.146
Mental component score	48.7 (47.5, 50.0)	50.2 (48.3, 52.1)	48.4 (45.8, 51.1)	0.376
HADS, mean (95% CI)^e^				
Anxiety scale	7.3 (6.8, 7.8)	6.7 (5.9, 7.5)	6.8 (5.8, 7.7)	0.306
Depression scale	5.7 (5.2, 6.1)	4.8 (4.1, 5.5)	5.8 (5.0, 6.7)	0.106
Risk factors				
Age, mean (95% CI) years	63.9 (63.1, 64.8)	66.1 (64.6, 67.7)	67.3 (65.4, 69.2)	0.002
BMI, mean (95% CI) kg/m^2^	29.9 (29.3, 30.5)	30.1 (29.1, 31.2)	32.5 (31.2, 33.8)	0.002
Female sex	177; 0.52 (0.47, 0.57)	62; 0.54 (0.45, 0.63)	59; 0.77 (0.67, 0.86)	0.001
Manual occupational class	173; 0.54 (0.48, 0.59)	55; 0.51 (0.41, 0.60)	44; 0.61 (0.50, 0.72)	0.406
Attended higher education	95; 0.28 (0.23, 0.33)	25; 0.24 (0.16, 0.32)	17; 0.23 (0.13, 0.32)	0.468
Previous ever foot or ankle injury	234; 0.68 (0.64, 0.73)	76; 0.66 (0.57, 0.75)	53; 0.70 (0.59, 0.80)	0.836
High‐/very high–heeled footwear ever worn between ages 20 and 49 years^f^	124; 0.71 (0.64, 0.78)	48; 0.79 (0.68, 0.89)	42; 0.71 (0.60, 0.83)	0.507
Narrow/very narrow toe box ever worn between ages 20 and 49 years^g^	201; 0.62 (0.58, 0.66)	76; 0.67 (0.59, 0.74)	56; 0.61 (0.51, 0.70)	0.493
Hallux valgus present in either foot^h^	133; 0.40 (0.35, 0.46)	56; 0.48 (0.40, 0.57)	44; 0.52 (0.41, 0.63)	0.105
Intermittent claudication^i^	26; 0.09 (0.06, 0.12)	6; 0.06 (0.01, 0.10)	3; 0.05 (0.00, 0.10)	0.388
Diabetes mellitus (type 2) or IFG	44; 0.14 (0.10, 0.18)	16; 0.14 (0.08, 0.20)	17; 0.22 (0.13, 0.32)	0.178
Hypertension	101; 0.33 (0.28, 0.38)	35; 0.31 (0.22, 0.39)	24; 0.29 (0.19, 0.38)	0.713
Dyslipidemia (raised cholesterol or triglycerides)	178; 0.56 (0.51, 0.61)	67; 0.60 (0.51, 0.69)	47; 0.62 (0.51, 0.73)	0.565
Metabolic syndrome^j^	60; 0.20 (0.15, 0.24)	20; 0.17 (0.11, 0.24)	21; 0.26 (0.16, 0.36)	0.303
Hip pain in last year	186; 0.56 (0.51, 0.61)	64; 0.55 (0.46, 0.64)	52; 0.64 (0.53, 0.75)	0.434
Knee pain in last year	249; 0.74 (0.69, 0.79)	87; 0.76 (0.68, 0.84)	69; 0.88 (0.81, 0.96)	0.050
Nodal hand OA^k^	68; 0.21 (0.17, 0.25)	26; 0.22 (0.15, 0.29)	31; 0.34 (0.24, 0.44)	0.040

a Values are the number; *P* (95% confidence interval [95% CI]) unless indicated otherwise. OA = osteoarthritis; MTP = metatarsophalangeal; MFPDI = Manchester Foot Pain and Disability Index; SF‐12 = Short Form 12 health survey; HADS = Hospital Anxiety and Depression Scale; BMI = body mass index; IFG = impaired fasting glucose.

b Adjusted for age and sex.

c Positive scores on the Rasched MFPDI indicate more pain and functional impairment 15.

d Lower scores on the SF‐12 indicate poorer physical and mental health 16.

e Higher scores on the HADS indicate more severe anxiety and depression 17.

f Exposure was restricted to females and defined as previous high or very high footwear worn on most days for at least one 10‐year period between ages 20 and 49 years.

g Defined as previous narrow or very narrow toe box footwear worn on most days for at least one 10‐year period between ages 20 and 49 years.

h Hallux valgus was determined by self‐report from line drawings of each foot that depicted increasing grades in the hallux valgus angle of 15^°^ 18.

i Intermittent claudication was defined as calf pain when walking at an ordinary pace on level ground or uphill (or when hurried) that disappears in ≤10 minutes by standing still 19.

j Metabolic syndrome was defined as the presence of ≥3 of the following; BMI >30kg/m^2^, hypertension, dyslipidemia, and type 2 diabetes mellitus or IFG.

k Nodal hand OA was defined as Kellgren/Lawrence grade ≥2 in ≥2 interphalangeal joints (rays 2–5) and Heberden or Bouchard nodes (rays 2–5) across either hand 30.

## DISCUSSION

Our findings, based on the pattern of joint involvement on plain radiography and comparative symptom and risk profiles, suggest a distinction between isolated first MTP joint OA and a form of more widespread OA in the foot that involves multiple midfoot joints. This latter group had more severe pain and disability and was associated with female sex and the presence of nodal hand OA. Our study found few other significant differences between these groups after adjusting for age and sex, although the range of information gathered on risk factors was relatively limited.

While patterning of OA in the foot has not been examined before in detail, our findings are consistent with previous observations that foot OA seems to affect multiple joints 6, and co‐occurrence is present in certain midfoot joints 31. The involvement of multiple foot joints is akin to the polyarticular and highly symmetrical form of OA that is seen in hands 3, 32. Although studies of symmetry in the hands have reported associations between the presence of OA in a joint and its presence in the same joint on the opposite hand 33, 34, 35, these studies only adjusted for age. We found comparable estimates for foot OA symmetry when adjusting for age alone, which then attenuated considerably when further adjustment was made for sex, foot joint, and total number of affected foot joints. We have previously shown a nearly 4‐fold increase in odds for hand OA symmetry in a parallel community‐based cohort 36. It appears, therefore, that OA in the weight‐bearing small joints of the feet demonstrates the same high level of symmetry as hand OA.

The identification of a subgroup with isolated first MTP joint involvement frequently occurring in isolation is suggestive that some individuals have a specific predilection for the development of OA in this joint, possibly as a result of altered foot structure or inappropriate footwear. Indeed, cross‐sectional studies have reported characteristic variations of skeletal morphology in 2 conditions commonly associated with first MTP joint OA: hallux valgus and hallux rigidus 37, 38. Although we found no significant differences in the prevalence of hallux valgus between the 3 subgroups, the role of other structural characteristics (such as variation in metatarsal length) cannot be discounted. Although non‐statistically significant, there was a slight increase in the probability that individuals had worn high‐ or very high–heeled shoes between the ages of 20 and 49 years, which is consistent with a previous study that found high‐heeled footwear to be associated specifically with disorders of the forefoot and toes 39. However, while the proportion reporting they had worn narrow‐toed footwear was higher in the isolated first MTP joint OA than the no or minimal foot OA subgroup, it was lowest in the polyarticular foot OA subgroup.

While multiple joint involvement and symmetry were observed in both the isolated first MTP joint and polyarticular foot OA subgroups, those in the polyarticular foot OA subgroup had wider joint involvement, which also included the first MTP joint. This is suggestive of a stronger influence of systemic risk factors and could be indicative of a generalized form of OA. The significantly greater proportion of females in the polyarticular foot OA subgroup is consistent with the strong patterns of symmetry and multiple joint involvement that has been seen in hand OA 32, 33. This has been ascribed to postmenopausal changes, increasing the susceptibility of females to the development of generalized OA 40. The significantly increased frequency of nodal OA in the polyarticular foot OA group would support the possible involvement of OA at other sites.

Metabolic factors have been associated with OA at other weight‐bearing 19, 41 and non–weight‐bearing sites 20, 42, through altered lipid metabolism and chronic inflammatory responses 43, 44. However, in this analysis only, increased BMI was found in those with polyarticular foot OA compared to the other subgroups. Alternatively, the increased BMI in the polyarticular foot OA could be indicative of a mechanical cause. Other research has found both obesity and alterations in midfoot loading to be associated with midfoot OA 45, 46.

The polyarticular foot OA subgroup was distinct cross‐sectionally from the other 2 classes of foot OA in terms of descriptive characteristics and symptoms, while differentiation between those classified as having no or minimal foot OA and those with isolated first MTP joint OA was negligible. However, minor differences included the isolated first MTP joint subgroup being slightly older, having more joints affected with radiographic OA, and having foot pain for slightly longer durations. These factors may represent the accumulation of joints affected by OA over time, and it's possible that isolated first MTP joint OA is a precursor to the development of more widespread foot OA seen in the polyarticular OA subgroup. Such progression might occur due to the modification of local biomechanical factors as a consequence of first MTP joint OA 47, altered foot biomechanics related to the presence of OA at the knee 48, or systemic factors as part of a generalized form of OA 40. However, the polyarticular foot OA subgroup were not found to be older than the isolated first MTP joint subgroup. Longitudinal data would be required to investigate this further.

While negligible differences in the symptom and risk factor profiles between the first MTP joint OA and the no or minimal foot OA subgroups do not confirm a distinct first MTP joint OA phenotype, its existence cannot be ruled out. The limited person‐level measures included in the analysis may have meant that discrimination was not possible. More comprehensive foot‐specific data on symptoms and risk factors, such as the type and location of foot injuries and objective functional measures, might be more informative. In addition, further insight into foot OA phenotypes will be achieved through replication of this work in different study populations, investigation of the clinical presentations, co‐occurrence of OA at other joints sites, and the course of symptoms over time.

The variation in symptoms along with the potentially different causal mechanisms indicates that separate treatment strategies may be appropriate. To date, a range of treatment options have been investigated for foot OA, including steroid joint injections 49, 50, insoles 51, and a range of surgical procedures 52, but the effectiveness of these treatments, in general 18 and particularly in relation to different forms of foot OA, is not known.

Several methodological strengths and limitations should be considered when interpreting the findings in this article. This analysis included participants recruited from the general population who reported having foot pain in the previous year; therefore a wide range of foot pain and radiographic severities were present. A standardized radiographic protocol was used to obtain weight‐bearing views so JSN was appropriately assessed, and multiple planes captured OA features, which have been noted to vary on different views 6. However, in this analysis only 5 joints in each foot were examined. It is possible that other foot joints may be affected by OA and contribute to the patterning and subgroups observed. Intrarater reliability for the presence of OA was found to be excellent. Despite interrater reliability being moderate, it was comparable with the original atlas 5. Although the study population had a prevalence of OA in 1 or more foot joints of 63%, when multiple foot joints were examined the numbers in some of the combinations were quite small. This is likely to have reduced the statistical power, potentially leading to type II error. Additionally, although all individuals in the study had reported having foot pain in the last year, the latent classes of foot OA were based only on radiographic structural changes. As discordance between symptoms and structural changes are often seen, further investigation characterizing polyarticular foot OA and first MTP joint OA in relation to symptomatic radiographic disease is needed.

In conclusion, this is the first detailed analysis of the pattern of multiple‐joint involvement in foot OA. We have demonstrated that, as is the case for OA at other small joint sites (particularly the hands), patterning of individual joint involvement in radiographic foot OA is polyarticular and strongly symmetrical. Patterns of joint involvement in radiographic foot OA have indicated a distinction between individuals with isolated first MTP joint OA and those with a more widespread form of OA labeled “polyarticular foot OA,” but that also includes one or both first MTP joints. Our findings of these different forms of foot OA have provided new insights into possible causes, with a joint‐specific predilection to OA at the first MTP joint and possible systemic risk factors and mechanical mechanisms, which leads to a more generalized presentation of OA that includes the midfoot. While a greater symptomatic burden was seen in those with polyarticular foot OA, further investigation is needed to examine if these subgroups differ in their foot‐specific symptoms, clinical presentation, and the symptomatic course over time to extend our understanding of foot OA and how it should be best managed.

## AUTHOR CONTRIBUTIONS

All authors were involved in drafting the article or revising it critically for important intellectual content, and all authors approved the final version to be submitted for publication. Ms Rathod had full access to all of the data in the study and takes responsibility for the integrity of the data and the accuracy of the data analysis.

### Study conception and design

Rathod, Marshall, MJ Thomas, Menz, Myers, Peat, Roddy.

### Acquisition of data

Marshall, MJ Thomas, Myers.

### Analysis and interpretation of data

Rathod, Marshall, MJ Thomas, Menz, Myers, E Thomas, Downes, Peat, Roddy.

## Supporting information


**Supplementary Figure 1.** A diagram illustrating the five foot joints examined and the frequency of radiographic OA in 533 adults aged 50 years and over *first MTP joint, first metatarsophalangeal joint; first CMJ, first cuneometatarsal joint; second CMJ, second cuneometatarsal joint; NCJ, navicular first cuneiform joint; TNJ, talonavicular joint*.

**Supplementary Figure 2.** A 5‐way Venn diagram showing the different combinations of joint involvement within the foot of all individuals (1066 feet) *first MTP joint, first metatarsophalangeal joint; first CMJ, first cuneometatarsal joint; second CMJ, second cuneometatarsal joint; NCJ, navicular first cuneiform joint; TNJ, talonavicular joint*.

Supplementary Table 1. Latent class characteristics for radiographic OA in different foot joints
