# Supplementary figures and images for: Investigations of Potential Phenotypes of Foot Osteoarthritis: Cross‐Sectional Analysis From the Clinical Assessment Study of the Foot
Source: Arthritis Care Res (Hoboken). 2016 Jan 25;68(2):217–27. doi: 10.1002/acr.22677 (PMC4819686; doi:10.1002/acr.22677)

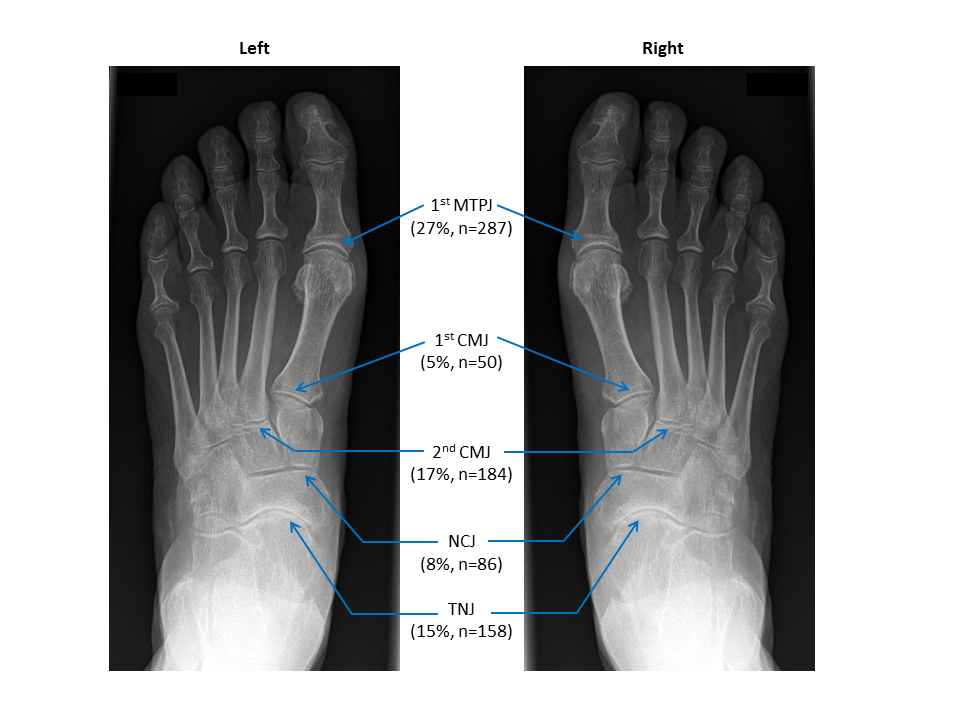

Supplement: Supplementary file 1 — Supplementary Figure 1. A diagram illustrating the five foot joints examined and the frequency of radiographic OA in 533 adults aged 50 years and over first MTP joint, first metatarsophalangeal joint; first CMJ, first cuneometatarsal joint; second CMJ, second cuneometatarsal joint; NCJ, navicular first cuneiform joint; TNJ, talonavicular joint. [file ACR-68-217-s002.tif]

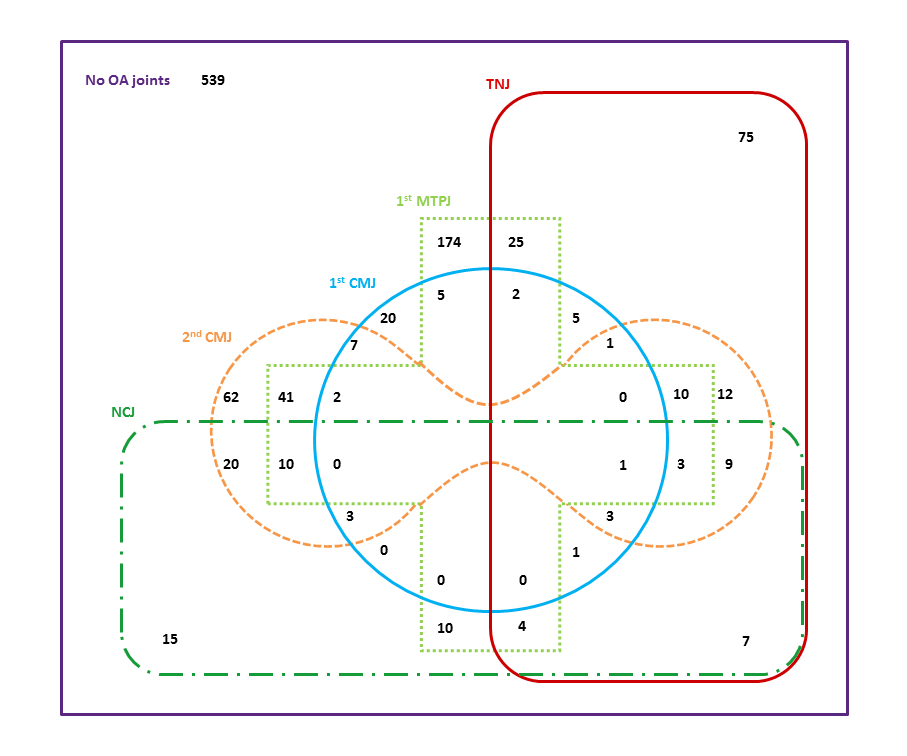

Supplement: Supplementary file 2 — Supplementary Figure 2. A 5‐way Venn diagram showing the different combinations of joint involvement within the foot of all individuals (1066 feet) first MTP joint, first metatarsophalangeal joint; first CMJ, first cuneometatarsal joint; second CMJ, second cuneometatarsal joint; NCJ, navicular first cuneiform joint; TNJ, talonavicular joint. [file ACR-68-217-s003.tif]
