# Supplementary material for: Investigations of Potential Phenotypes of Foot Osteoarthritis: Cross‐Sectional Analysis From the Clinical Assessment Study of the Foot
Source: Arthritis Care Res (Hoboken). 2016 Jan 25;68(2):217–27. doi: 10.1002/acr.22677 (PMC4819686; doi:10.1002/acr.22677)
Supplement: Supplementary file 3 — Supplementary Table 1. Latent class characteristics for radiographic OA in different foot joints [file ACR-68-217-s001.docx]

Supplementary Table 1. Latent class characteristics for radiographic OA in different foot joints

| Number of classes | AIC | BIC | Sample size adjusted BIC | Lo-Mendell-Rubin adjusted LRT  P value | Entropy |
| --- | --- | --- | --- | --- | --- |
| 1 | 4125.5 | 4168.3 | 4136.5 | - | - |
| 2 | 3915.6 | 4005.5 | 3938.8 | <0.001 | 0.73 |
| **3** | **3804.2** | **3941.1** | **3839.5** | **<0.001** | **0.87** |
| 4 | 3772.7 | 3956.7 | 3820.2 | 0.076 | 0.88 |
| 5 | 3759.0 | 3990.1 | 3818.6 | 0.131 | 0.82 |
| 6 | 3751.0 | 4029.1 | 3822.8 | 0.255 | 0.87 |

*AIC, Akaike Information Criteria; BIC, Bayesian Information Criteria; LRT, likelihood ratio test. The bold text indicates the model that was selected as having the optimal number or classes. Lower AIC and BIC, and higher entropy values indicate the optimal number of classes. The change in p-value for the Lo-Mendell-Rubin adjusted LRT from being significant to non-significant indicates where an additional class does not improve the latent class model.*
